# RFID Ownership Transfer with Positive Secrecy Capacity Channels

**DOI:** 10.3390/s17010053

**Published:** 2016-12-29

**Authors:** Jorge Munilla, Mike Burmester, Alberto Peinado, Guomin Yang, Willy Susilo

**Affiliations:** 1 Escuela Técnica Superior de Ingenieros de Telecomunicación, Universidad de Málaga, Málaga 29071, Spain; apeinado@ic.uma.es; 2 Department of Computer Science, Florida State University, Tallahassee, FL 32306, USA; burmester@cs.fsu.edu; 3 School of Computer Science and Software Engineering, University of Wollongong, Wollongong, NSW 2522, Australia; gyang@uow.edu.au (G.Y.); wsusilo@uow.edu.au (W.S.)

**Keywords:** RFID, ownership transfer, trusted third party, RFID, EPCglobal Gen2

## Abstract

RFID ownership transfer protocols (OTPs) transfer tag ownership rights. Recently, there has been considerable interest in such protocols; however, guaranteeing privacy for symmetric-key settings without trusted third parties (TTPs) is a challenge still unresolved. In this paper, we address this issue and show that it can be solved by using channels with positive secrecy capacity. We implement these channels with noisy tags and provide practical values, thus proving that perfect secrecy is theoretically possible. We then define a communication model that captures spatiotemporal events and describe a first example of symmetric-key based OTP that: (i) is formally secure in the proposed communication model and (ii) achieves privacy with a noisy tag wiretap channel without TTPs.

## 1. Introduction

Radio frequency identification (RFID) is a widely-deployed technology for supply-chain and inventory management, retail operations and more generally automatic identification. Most of these applications need to be secured.

Ownership transfer protocols (OTPs) allow the secure transfer of tag ownership from a current owner to a new owner. Three different entities are present in an OTP: the tag T whose rights are being transferred, the current owner who has the initial control of T and the new owner who will take control of T when the protocol is completed. OTPs must incorporate security requirements that protect the privacy of both the new and the previous owner of the tag. For RFID applications privacy addresses anonymity that protects the identity of tags and untraceability that prevents interrogations (partial or completed) of a tag being linked. Formal definitions for secure ownership and ownership transfer are provided by van Deursen et al. [1], while several theoretical models have been proposed in the literature that address the privacy of RFID systems [2,3,4,5].

Several OTPs that address security issues have been proposed. However, preventing a previous owner from accessing the key(s) of a tag whose ownership was transferred is still an unsolved problem when symmetric-key techniques are used [6,7]. The current approach for privacy is to either employ a trusted third party (TTP) to break the trust link between a tag and its owner (e.g., [8,9]), or an isolated environment (ISE) (e.g., [10,11]) without any adversarial interference. The first approach is centralized and not appropriate when tags belong to different authorities/companies. In fact, the TTP can be considered as the real holder of the tag’s rights, while the different owners have simply delegated ownership. The second approach assumes a weak threat model and, as claimed in [7]: if such protection is adequate, then there is no need for security. Our main contributions in this paper are to:

(1) Define a communication model for ownership transfer that addresses spatiotemporal connectivity (Section 3). Many OTPs do not specify the communication setup and assume channels that are impractical for RFID settings.
(2) Provide a theoretical analysis of wiretaps with noisy tags (Section 4), show how these could be implemented and prove that perfect secrecy is achievable.
(3) Present an OTP that is provably secure in this communication model and that uses a wiretap channel with noisy tags to achieve privacy (Section 5). This is the first example of symmetric-key-based OTP that does not require TTPs or an ISE. GNYlogic and strand spaces [12,13,14,15] are used in the Appendix A for the security analysis.

## 2. Background

### 2.1. Definition and Security Requirements

Tag ownership can be defined as the ability to identify and/or access the tag, which in turn usually implies knowledge of private keys stored on the tag. Ownership transfer protocols enable the transfer of ownership rights of a tag T from the current owner ${\mathrm{Own}}_{c}$, or seller, to a new owner ${\mathrm{Own}}_{n}$ or buyer. At the beginning of the OTP, the seller is the only entity that can identify and trace T, while when the OTP is completed, T can only be identified and/or traced by the buyer. A TTP is usually deployed to manage this ownership transfer.

We next list some specific security requirements for OTPs:

Unlinkability or untraceability. An adversary that physically tracks tags can easily determine which executions are linked. This cannot be prevented. Unlinkability is related to the capability of linking interrogations after this physical tracking is temporarily interrupted. Different formal models can be found in the literature (e.g., [2,3,4]). Intuitively, a protocol guarantees unlinkability or privacy if no adversary can decide with advantage better than negligible whether two messages taken from different protocol executions belong to the same tag or not.

Privacy of ${\mathrm{Own}}_{n}$ (backward secrecy): The current owner ${\mathrm{Own}}_{c}$ cannot identify T once ownership rights are transferred to the new owner ${\mathrm{Own}}_{n}$.

Privacy of ${\mathrm{Own}}_{c}$ (forward secrecy): Once ownership rights of T are transferred to the new owner ${\mathrm{Own}}_{n}$, past communications between T and previous owners cannot be traced by an adversary (or subsequent owners), even if the current private information stored on T is revealed (e.g., by physical attacks).

OTPs are sometimes designed [10,16,17] to provide extended capabilities such as: tag assurance, undeniable ownership transfer, current ownership proof, ownership delegation and authorized recovery.

### 2.2. Related Work

We only review the most relevant symmetric-key-based OTPs for RFID. Saito et al. [18] and Molnar et al. [16] presented in 2005 the first OTPs for RFID applications. Saito et al. proposed two protocols: one with and one without TTP. The security of the latter is based on the short range of the backward channel and assumes that it is hard for adversaries to eavesdrop on this channel. Molnar et al. proposed a scheme with TTP to manage tag keys by using a tree structure. Some vulnerabilities of this scheme are discussed in [19]. Soppera and Burbridge [20] modified Molnar et al.’s scheme by replacing the TTP with distributed local devices called RFID acceptor tags. Osaka et al. [21] used a kind of TTP with hash values to protect messages and a keyed encryption function for ownership transfer. Chen et al. [22] and Japinnen and Hamalainen [23] modified Osaka et al.’s scheme to prevent DoS attacks. Yoon and Yoo [24] also modified Osaka et al.’s scheme, by assuming that owners are able to change the tag’s key in an ISE. Their scheme had some vulnerabilities described in [25]. Dimitriou [26] proposed RFIDdot, an ownership transfer scheme based on random nonces and a keyed encryption function, making the assumption that key updates are performed in a private environment. More recently, Song and Mitchell [27,28] also assumed an ISE, but used keyed hash functions and one-time tag identifiers with hash chains. Kapoor and Piramuthu proposed two new schemes [7] based on a TTP and ISE respectively for the transfer of single tags, while a variant of these protocols for multiple tags has also been published [29]. Finally, several schemes have recently been proposed that comply with the EPCGen2 [30] standard for low-cost tags in the UHF band. These again assume TTPs or ISE and combine simple XOR operations, Cyclic Redundancy Codes (CRC16) and/or use the on-board PRNG as the security primitive (e.g., [9,31,32,33]). The security problems of some of these have been described recently [34].

#### Motivation: Comparison with Previous Works

As observed, the ownership transfer protocols proposed in the literature rely either on the use of TTPs or the assumption of an ISE. Typically, TTPs have a centralized management that may not be compatible with the distributed management of RFID systems. For example, the RFID parties (the owners) with possibly conflicting interests must trust the TTP that manages their tags. On the other hand, the assumption of ISEs where no adversary can interfere is an assumption of a weak adversary model: if such an environment were available, then no other security protection would be needed [7]. This paper proposes a key exchange protocol that addresses the new owner’s privacy concerns without resorting to either TTPs or an ISE.

**Figure 1:**
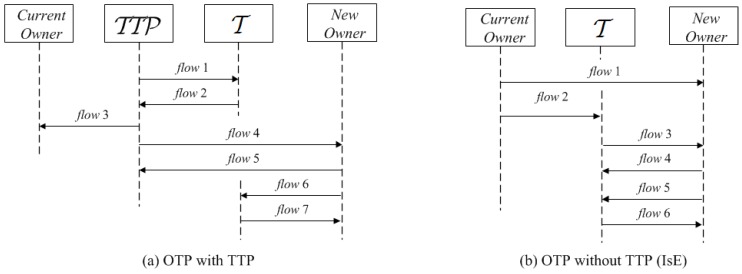
Example sketches of ownership transfer protocols (OTPs) with trusted third parties (TTPs) (**a**) and without TTPs (isolated environment) (**b**) [7].

The discussed protocols also use communication models that are sometimes impractical for real-life scenarios. To illustrate this, let us consider the two protocols proposed in [7]: one with TTP, the other without TTP (but with an ISE), whose flows are shown in Figure 1. In the first, Figure 1a, the TTP does not use a reader to communicate with tag T, but communicates directly (Flows 1–2). This begs the question: if such a TTP were installed in the buyer’s or seller’s location, what trust issues would arise if the transferred goods belong to different authorities. In the second protocol, Figure 1b, T interacts first with the current owner (the seller, Flow 2) and then with the new owner (the buyer, Flows 3–6). However if something goes wrong (Flow 6 is not received correctly), then the process must be repeated from the beginning. This implies that the buyer and the seller must be available during the transaction, which restricts the possible transaction scenarios to one location (e.g., to a shop). In this paper, we define a communication model where tags can only communicate through readers. This leads to designs of protocols with, if deployed, centralized TTP infrastructures and, in contrast to the examples described above, that allow the seller and buyer to be in different physical locations.

## 3. A Communication Model for RFID Ownership Transfer

### 3.1. Entity Capabilities

High-level entities include RFID readers, servers and TTPs. In general, these are able to perform complex cryptographic operations, such as asymmetric encryption/decryption and digital signatures/verification.

RFID tags: In this paper, we are only concerned with UHF passive tags that operate in the far field [35], which are the most common for supply chain applications. These work at higher distances than tags with inductive coupling, but the delivered power is low; therefore, not too complex (lightweight) cryptographic tools should be used [36]. Low price is also a common requirement, and therefore, tamper-resistant shielding and on-board clocks cannot be usually assumed.

### 3.2. Communication Model

This is defined in terms of its channels with security features, such as privacy and integrity, and connectivity (availability).

#### 3.2.1. Privacy/Integrity Channels

Between high-level entities (readers, servers or TTPs): These can be considered secure, since fully-fledged cryptographic techniques can be used.

Between readers and tags: By contrast, these are particularly vulnerable; they are wireless (the adversary can eavesdrop and block/modify/inject messages), and tags can only implement lightweight cryptographic mechanisms. Passive tags can only communicate with active entities that are physically close and provide them with energy: i.e., RFID readers.

#### 3.2.2. Connectivity

**Figure 2:**
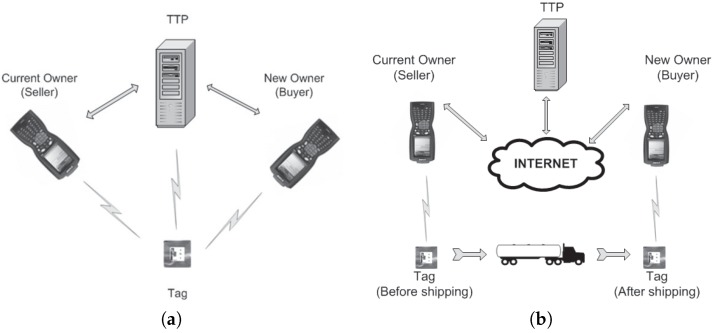
OTP communication models. (**a**) Basic model (static); (**b**) Dynamic model.

Connectivity is a function of space and time. As far as we know, OTPs proposed in the literature do not discuss spatiotemporal connectivity issues, though several ( e.g., [7,9,17]) assume channels that allow high-level parties, including a TTP (e.g., [7]), to communicate with a tag T in real time during the execution of the OTP: for example, to restart the protocol if it fails. This implies that T must be physically close to the corresponding high-level parties during the execution of the protocol, which in many practical scenarios may not be the case. Suppose for example that a client purchases RFID-tagged items for tracking and counterfeit prevention via the Internet. The seller dispatches the items, and when these reach the destination, the client requests the transfer of ownership rights. In this case, ownership transfer takes place in a different location from the seller’s location, and a different connectivity model is needed, where the seller cannot communicate with the tags at this stage (likewise, buyers cannot communicate with tags at the beginning of the transaction). We also need a spatiotemporal TTP network infrastructure in which TTPs may have to communicate in real time (as in [7]). Figure 2 illustrates the differences between the traditional and the extended communication model.

Let R1, R2, TTP be the readers of ${\mathrm{Own}}_{c}$, ${\mathrm{Own}}_{n}$, TTP, T a tag, a,b be OTP parties and $\exists \;(a\overset{\;t}{↔}b)$, $\exists \;(a\overset{\;t}{\Leftrightarrow }b)$ stand for “there exists a channel at time *t* between a,b”, “there exists a secure channel at time *t* between a,b”, respectively. When *t* is not indicated, continuous connectivity is assumed. We formally define the connectivity requirements of the OTP model by the relations:

1. ∃(R1⇔R2)∧∃(R1⇔TTP)∧∃(R2⇔TTP),
2. $\left.\begin{array}{c} \exists \;\left(R1\overset{t}{↔}T\right)\;\;\mathrm{for}\;t_{0}\leq t<t_{1}\\ \exists \;\left(R2\overset{t}{↔}T\right)\;\;\mathrm{for}\;t_{2}\leq t<t_{3} \end{array}\right\}\;\;\mathrm{with}\;t_{1}\leq t_{2}$.

Thus, a TTP, if deployed, can only communicate with tags T via readers R1, R2.

## 4. A Wiretap Channel with Positive Secrecy Capacity

To guarantee the privacy of a new owner ${\mathrm{Own}}_{n}$ of a tag T and prevent the previous owner ${\mathrm{Own}}_{c}$ from accessing T, ${\mathrm{Own}}_{n}$ and T must agree on a fresh key in the presence of ${\mathrm{Own}}_{c}$: that is, with ${\mathrm{Own}}_{c}$ a potential eavesdropper. Note that ${\mathrm{Own}}_{c}$ has full knowledge of the private keys of T. We shall show that by using Wyner’s wiretap channel [37] with noisy tags, we can achieve positive secrecy.

The fundamental property of the superposition of the wireless medium can be pitted against eavesdropping by using interference at the physical layer to degrade communication. Degrading is implemented via reader-controlled interferers called noisy tags. Noisy tags were first used by Juels et al. [38] to protect consumers from unwanted RFID scanning. Later, Castellucia and Avoine [39] used noisy tags for sharing secret keys, which however only addresses passive adversaries since authentication is not ensured. We shall assume that noisy tags do not present any special features, so any tag can become a noisy tag. If more sophisticated noisy tags are available, then implementations with better performance can obviously be achieved.

**Figure 3:**
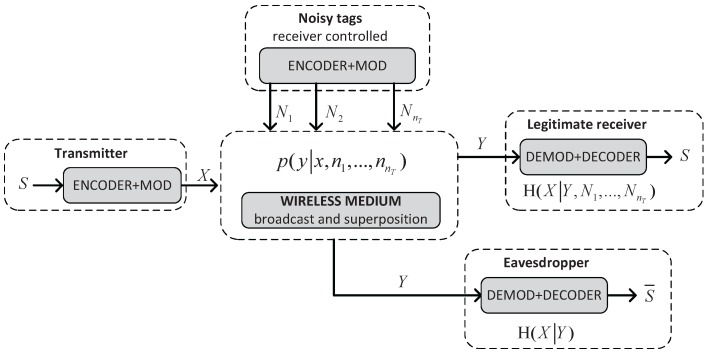
A model for the wiretap channel with noisy tags.

We use the following notation: X,Y,N are random variables taking values x,y,n in the alphabets X,Y,N, respectively. Figure 3 depicts our model of a wiretap channel with input alphabets $X,N_{1},\ldots ,N_{n_{T}}$, output alphabet Y and transition probabilities $p(y|x,n_{1},\ldots ,n_{n_{T}})$.

Tag T transmits the message *S* (coded as *X*) to the new owner ${\mathrm{Own}}_{n}$ (the intended receiver) with the help of $n_{T}$ noisy tags, in the presence of the current owner ${\mathrm{Own}}_{c}$, who acts as a passive eavesdropper. The wiretap channel can be seen as a stochastic encoder of *X* with output alphabet Y. The variable *Y* is input to the maximum a posteriori probability (MAP) estimators of ${\mathrm{Own}}_{n}$ and ${\mathrm{Own}}_{c}$, but while ${\mathrm{Own}}_{c}$ only knows the value of *Y*, ${\mathrm{Own}}_{n}$ also knows the values of the inputs $N_{1},\ldots ,N_{n_{T}}$. Thus, if we assume the wireless medium is noiseless, then the estimate S=s of ${\mathrm{Own}}_{n}$ is correct, while the estimate $\bar{S}=\bar{s}$ of ${\mathrm{Own}}_{c}$ is degraded by the stochastic encoder. This degradation can be quantified by the conditional entropy H(X|Y).

(1) $$H\left(X|Y\right)=\sum_{j=0}^{|X|-1}\sum_{k=0}^{|Y|-1}-p\left(x_{j},y_{k}\right)\cdot \log_{2}p\left(x_{j}|y_{k}\right)$$

**Figure 4:**
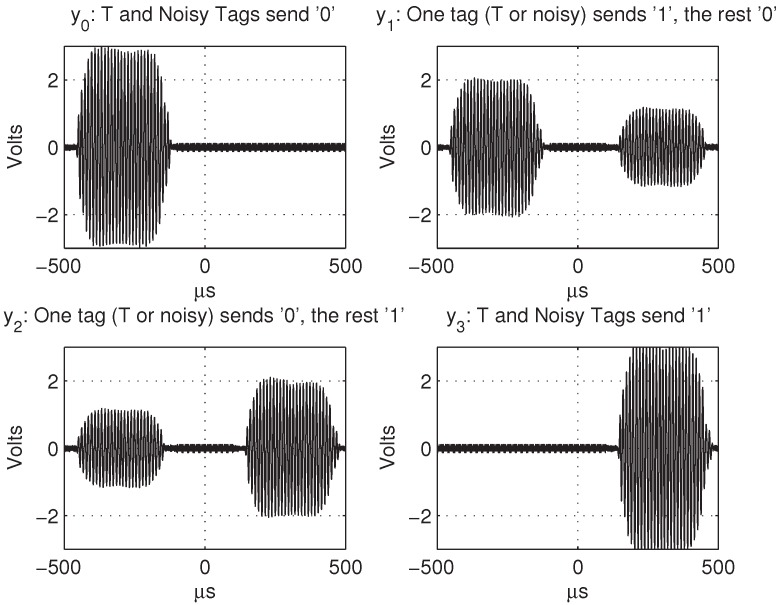
Alphabet $Y=\{y_{0},y_{1},y_{2},y_{3}\}$ for tag T and two noisy tags using pulse position modulation (PPM).

The capacity of the eavesdropper channel (${\mathrm{Own}}_{c}$’s) is defined as $C_{eav}=H\left(X\right)-H\left(X|Y\right)$. The secrecy capacity for the wiretap model is $C_{s}=C_{\mathrm{main}}-C_{eav}$, where $C_{\mathrm{main}}$ is the capacity of the main channel (${\mathrm{Own}}_{n}$’s). In the noiseless case, we have $C_{\mathrm{main}}=H\left(X\right)$, and therefore, the secrecy capacity coincides with the conditional entropy of the eavesdropper $C_{s}=H\left(X|Y\right)$, while the analysis of secrecy reduces to the eavesdropper’s channel. In general, the more degraded the wiretap channel, the higher the secrecy capacity. We assume for this analysis that the adversary cannot identify the source of each message via signal characteristics (fingerprints, level power, phase shifts, etc.). This implies that tags should be close and implement the same modulation alphabet; i.e., $N_{j}=X$, $1\leq j\leq n_{T}$. Possible implementation imperfections, such as delays, signal levels, frequency deviations, etc., should not reveal their origin; i.e., be insignificant or have sufficient randomness. Note that this assumption is implicit in the RFID literature in protocols that address privacy issues: traceability cannot be prevented if tags are physically identified. In this particular case, to prevent an adversary from identifying the target tag, we should guarantee that the tag is close enough to the noisy tags and that it does not present distinguishable imperfections; i.e., insignificant or significant, but changing in every execution. In practice, fortunately, although it is true that no two tags have identical signals, the differences are typically insignificant, making it hard to disambiguate them. As a consequence of the superposition property of the wireless channel, from a theoretical point of view, any modulation can be used (with initial calibration if required), but in practice, some modulations have better features than others. Figure 4 shows a simplified example that uses PPM (pulse position modulation). A bit is encoded by transmitting a pulse in one of two possible time slots. Synchronization between tags is helped by the fact that they share the same reference (reader’s) signal. Perfect synchronization is not necessary: tags may have different delays provided there is no pattern that can be exploited to identify a tag.

If noise and imperfection implementations are not considered, the security of the system relies exclusively on the stochastic encoder. For *r*-ary input alphabets $X\;=\;\{x_{0},x_{1},...,x_{r-1}\}$, with $p(x_{i})\;=\;1/r$, 0 ≤ i ≤ r - 1, the output alphabet is $Y\;=\;{\left\{y_{i}\right\}}_{i=0}^{|Y|-1}$, and the cardinality of Y (combinations with repetition of *r* elements taken $n_{T}+1$ at a time) and the transition probabilities can be computed as follows:

(2) $$\left|Y\right|=\left(\begin{array}{c} n_{T}+r\\ r-1 \end{array}\right)=\left(\begin{array}{c} n_{T}+r\\ n_{T}+1 \end{array}\right),$$

(3) $$p\left(y_{m_{0}m_{1}...m_{r-1}}|x_{i}\right)=\frac{1}{r^{n_{T}}}\left(\begin{array}{c} n_{T}\\ m_{0}\;\;m_{1}\;...\;m_{r-1} \end{array}\right)$$

where $y_{m_{0}m_{1}...m_{r-1}}$ is the output symbol resulting from the combination of $m_{0}$ symbols $x_{0}$, $m_{1}$ symbols $x_{1}$, and so on, until $m_{r-1}$ symbols $x_{r-1}$, with $m_{0}\;+\;m_{1}\;+\;...\;+\;m_{r-1}\;=\;n_{T}$.

Particularizing for binary input alphabets (r = 2), $X\;=\;\{x_{0},x_{1}\}$, with $p\left(x_{0}\right)\;=\;p\left(x_{1}\right)\;=\;0.5$ (H(X)=1), the output alphabet is $Y\;=\;{\left\{y_{i}\right\}}_{i=0}^{n_{T}+1}$, where $y_{i}$ is the combination of *i* symbols $x_{0}$ and $\left(n_{T}\;+\;1\;-\;i\right)$ symbols $x_{1}$. The transition probabilities $p(y_{i}|x_{j})$ are given by:

(4) p(yi|x0)=p(yN+1-i|x1)=2-nTnTi,i=0,…,nT+1.

${\mathrm{Own}}_{c}$’s detector receives $y_{i}$ and applies the decoding specified by:

(5) $$\begin{array}{cc} \;n_{T}\;\mathrm{even},\bar{s}= & \left\{\begin{array}{c} \;g(x_{0})\;\mathrm{if}\;i<\frac{n_{T}+1}{2}\\ \;g(x_{1})\;\mathrm{otherwise} \end{array}\right.\\ n_{T}\;\mathrm{odd},\bar{s}= & \left\{\begin{array}{c} \;g(x_{0})\;\mathrm{if}\;i<\frac{n_{T}+1}{2}\\ \;g(x_{1})\;\mathrm{if}\;i>\frac{n_{T}+1}{2}\\ \;\mathrm{otherwise},\;\mathrm{choose at random}\;g(x_{0})\;\mathrm{or}\;g(x_{1}) \end{array}\right. \end{array}$$

with *g* the mapping function g:X → S.

**Figure 5:**
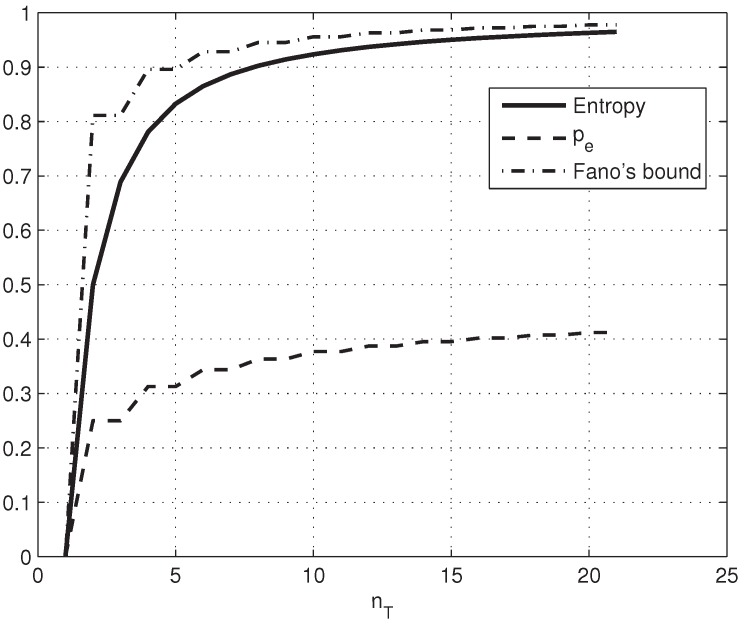
The conditional entropy, error and Fano’s bounds of the wiretap channel.

The error probability, defined as $p_{e}\;=\;Pr\;\left[\bar{s}\;\neq \;s\right]$, is computed as:

(6) pe=2-nT∑i=0nT2-1nTi+12nTnT+12,

where the last summand is zero when $n_{T}$ is even. Figure 5 plots the secrecy capacity $C_{s}$ of the wiretap channel, the error probability and Fano’s bound, against the number of noisy tags. Secrecy increases sharply until $n_{T}\;\approx \;5$; as $n_{T}\;\to \;\infty$, the equivocation of the eavesdropper approaches the unconditional source entropy, and we get perfect secrecy: $\lim_{n_{T}\to \infty }H\left(X|Y^{\left(n_{T}\right)}\right)\;=\;H\left(X\right)\;=\;1$. For $n_{T}\;=\;3$, the secrecy capacity $C_{s}\;=\;H\left(X|Y\right)\;=\;0.78$ offers a good compromise between features and ease of implementation. The capacity of ${\mathrm{Own}}_{c}$’s channel is just $C_{eav}\;=\;0.22$ bits.

## 5. An Ownership Transfer Protocol

We next present an example of an OTP that: (i) works according to the communication model defined in Section 3.2 and (ii) uses a channel with positive secrecy capacity, implemented with noisy tags, to guarantee the privacy of the new owner.

The protocol addresses practical design features, such as (secure) singulation of tags and the interrogator-talks-first requirement (communication must be initiated by the reader), and guarantees that the information stored on the tag coincides with that provided to the new owner (tag assurance [17]). Note also that it complies with the restrictions in Section 3.1 regarding entities’ capabilities. That is, while RFID readers can implement fully-fledged cryptographic tools, RFID tags are restricted to a pseudorandom number generator (PRNG) and a cryptographic (one-way, collision-resistant) hash function $F\;:\;{\left\{0,1\right\}}^{*}\;\to \;{\left\{0,1\right\}}^{n}$. The number of inputs is, however, designed to be intentionally low so that it can be more easily adapted to other possible primitives. We assume that identifiers, random numbers and keys all have the same (bit) length *n*, which is the security parameter of the protocol. We introduce our notation.

IDidentifying information of T.InfoIDhash of the manufacturer information.R1, R2readers of Ownc and Ownn respectively.IDR1,IDR2identifiers for R1 and R2 respectively.s1key that T shares with R1.s2key thatT shares with R2.s2¯key that T eventually shares with R2.NT, NT′random numbers generated by T.NR1random number generated by R1.NR2, NR2′random numbers generated by R2.Tt∗the t noisy tag,with 1 ≤ t ≤ nT.st∗the key that the Tt∗ shares with R2.

### 5.1. The Ownership Transfer Protocol, Figure 6

Initialization

1. Initially, each owner knows for each tag ID its information and private key $s_{1}$. Likewise, each tag stores, along with its identifier ID and ${\mathrm{Info}}_{\mathrm{ID}}$, the identifier of its owner IDR1 and the private key. R1, R2 agree to transfer ownership of tag T with identifier ID. R1 sends (secure channel) R2 manufacturer information about the tag (${\mathrm{Info}}_{\mathrm{ID}}$ when hashed).
  R1 ⇒ R2 : ID, manufacturer information

Setup for Ownership Transfer

2. R1 regularly broadcasts Query messages to detect the presence of tags.
  R1 → tags:Query
3. When T receives a Query (presumably because it is within the range of R1), it selects a random nonce $N_{T}$ and sends:
  $$T\;\to \;R1\;:\;F\left(N_{T},s_{1}\right),\;N_{T}$$
4. R1 searches for a pair (ID,s) in its database to get a match. If there is no match, then the process is repeated from Step 2. Otherwise, T is singulated: R1 selects a random nonce $N_{R1}$ and a request OTR and sends:
  $$R1\;\to \;T\;:\;\mathrm{OTR},\mathrm{IDR}1,\mathrm{IDR}2,F\left(s_{1},N_{T}\right),N_{R1}$$
5. T checks $F(s_{1},N_{T})$ to authenticate R1. T does not reply if there is no match. Otherwise, it computes $s^{\prime }\;=\;F\left(N_{T},N_{R1},s_{1}\right)$, saves [IDR2, $s^{\prime }]$, until the protocol completes or a new command from R1 is received and replies with:
  $$T\;\to \;R1\;:\;F(N_{R1},s_{1})$$
6. If this message is not received correctly by R1 after a period of time, the protocol is repeated from Step 2 (T will replace the stored values $\mathrm{IDR}2,s^{\prime }$). Otherwise, R1 computes $s^{\prime }\;=\;F\left(N_{T},N_{R1},s_{1}\right)$ and confirms (secure channel) to R2 that T is ready to be transferred:
  $$R1\;\Rightarrow \;R2\;:\;\mathrm{ID}\;\mathrm{is}\;\mathrm{ready},s^{\prime }$$

Ownership Transfer

7. If R2 receives R1’s confirmation, then it is ready to take ownership of T. R2 computes $s_{2}\;=\;F\left(s^{\prime },{\mathrm{Info}}_{\mathrm{ID}}\right)$ and broadcasts regularly Query messages.
  R2 → tags:Query
8. When T receives a Query, it selects a random nonce $N_{T}^{\prime }$ and sends:
  $$T\;\to \;R2\;:\;F\left(N_{T}^{\prime },s_{2}\right),N_{T}^{\prime }$$
9. If T is singulated, then R2 selects a fresh random number $N_{R2}$ and sends:
  $$R2\;\to \;T\;:F\left(s_{2},N_{T}^{\prime }\right),N_{R2}$$
10. T checks this message for $s_{2}$, and if not correct, for $s_{1}$ (and waits for new commands). It does not reply if this is not correct. If R2 is authenticated, T updates the stored values $\left(\mathrm{IDR}1,s_{1}\right)$ to $\left(\mathrm{IDR}2,s_{2}\right)$. These values determine tag ownership. T acknowledges this by sending:
  $$T\;\to \;R2:F(N_{R2},s_{2})$$
11. If the received message is not correct, the protocol is repeated from Step 7. Otherwise, R2 executes the key update protocol in Section 5.2 to prevent R1 from accessing T.

#### 5.1.1. Analysis

In the Appendix A, we shall use GNY logic [12], which extends the Burrows–Abadi–Needham (BAN) logic (overcoming some of its problems [13,14]), to show the consistency of the assumptions with respect to the source message, as well as the beliefs of the sender and receiver of messages. Principals can only advance their beliefs and increase their possessions based on the physical content of the messages they receive. We use strand spaces [15] to show correctness by excluding vulnerabilities based on the structure of the protocol. Strand spaces use free encryption algebra to detect faults that exploit relations in this algebra. Below, we discuss the most important security properties informally.

1 Untraceable singulation: Replies to Query’s (Step 2, Step 7) have the same format and include a nonce selected by the tag. This prevents tag tracing, since messages look random to anyone who does not know the secret key.
2 The privacy of ${\mathrm{Own}}_{c}$ is guaranteed because the key $s_{1}$ remains unknown to the new owner ${\mathrm{Own}}_{n}$. Indeed, if ${\mathrm{Own}}_{n}$ can compute $s_{1}$ given the values: $s^{\prime }$, $N_{T}$ and $N_{R1}$, then ${\mathrm{Own}}_{n}$ can also find the *F*-preimage of $s^{\prime }$, which contradicts the assumption that *F* is one-way.
3 Forward secrecy: Suppose the adversary succeeds in getting the new key $s_{2}$ of a tag. The privacy of the prior communications is guaranteed, as in the previous case, because to get $s_{1}$ from $s_{2}$, one has to invert *F*.
4 The privacy of ${\mathrm{Own}}_{n}$ is achieved by using the key update protocol in Section 5.2.
5 Tag assurance: ${\mathrm{Info}}_{\mathrm{ID}}$ is the hash of manufacturer information about the tag. The collision resistance of hash functions prevents the adversary from finding another message (pre-image) ${\mathrm{Info}}_{\mathrm{ID}}^{\prime }$ with the same hash to forge the information given by the manufacturer. The use of ${\mathrm{Info}}_{\mathrm{ID}}$ to compute $s_{2}$ guarantees that the information provided by ${\mathrm{Own}}_{c}$ to ${\mathrm{Own}}_{n}$ matches with the information stored by T. Note, however, that cloned tags and corruptible memories are beyond this security feature (cf. [17]).

### 5.2. A Key Update Protocol, Figure 7

The parties are: the reader R2, tag T and $n_{T}$ noisy tags $T_{t}^{*}$, $1\;\leq \;t\leq n_{T}$. R2 shares with T a private key $s_{2}$ and with each $T_{t}^{*}$ a private key $s_{t}^{*}$. In this protocol, T updates privately the key $s_{2}$ with a fresh key ${\bar{s}}_{2}$.

1 R2 broadcasts a key change request (KCR) with a random nonce $N_{R2}^{\prime }$.
  $$R2\;\to \;T,\;{\left\{T_{t}^{*}\right\}}_{t=1}^{n_{T}}:\;\mathrm{KCR},N_{R2}^{\prime }$$
2 Upon receiving this, T and $T_{t}^{*}$ generate bitstrings *S* and $S_{t}^{*}$ of length $n/C_{s}$ and broadcast these simultaneously (as specified in Section 4): *S* is a random number, and $S_{t}^{*}=F^{*}\left(N_{R2}^{\prime },s_{t}^{*}\right)$, where $F^{*}$ is a cryptographic hash function of length $n/C_{s}$. Note that $F^{*}$ could be built from *F*; for example, for $C_{s}=0.5$, $F^{*}\left(A,B\right)=F\left(A,B\right)\left|\right|F\left(A+1,B\right)$, where || denotes concatenation.
  $$T,\;{\left\{T_{t}^{*}\right\}}_{t=1}^{n_{T}}\;\to \;R2:\;S\;\mathrm{and}\;{\left\{S_{t}^{*}\right\}}_{t=1}^{n_{T}}$$
3 R2 receives the added signals of *S* and ${\left\{S_{t}^{*}\right\}}_{t=1}^{n_{T}}$, extracts *S*, computes ${\bar{s}}_{2}=F\left(N_{R2}^{\prime },S,s_{2}\right)$ and broadcasts $F(S,{\bar{s}}_{2})$.
  $$R2\;\to \;T,\;{\left\{T_{t}^{*}\right\}}_{t=1}^{n_{T}}:\;F\left(S,{\bar{s}}_{2}\right)$$
4 T computes ${\bar{s}}_{2}=F\left(N_{R2}^{\prime },S,s_{2}\right)$ and checks that the message from R2 is correct. If so, T updates its private key $s_{2}$ to ${\bar{s}}_{2}$.
  $$T\to R2:F(N_{R2}^{\prime },{\bar{s}}_{2})$$
5 R2 checks the received message. If correct, the key update protocol (KUP) is completed, and R2 informs R1. Otherwise, R2 sends a new Query and checks if T has updated its key. If not, the KUP is repeated.
  R2⇒R1:Ownershipistransferred.

### 5.3. Analysis

Attacks by external adversaries on the KUP can target privacy (traceability) or availability (de-synchronization). These are prevented by the wiretap channel with positive secrecy and a cryptographic hash function that authenticates messages. More specifically:

Traceability: T remains untraceable because the exchanged messages look random to anyone who does not know $s_{2}$.

De-synchronization: The adversary cannot compute $F(N_{R2}^{\prime },{\bar{s}}_{s})$ or $F(S,{\bar{s}}_{2})$, that are required by parties to update their keys, without knowing $s_{2}$.

The protection extends to threats from past and future owners of T. For example, even if R1 knows $s_{1}$ and can get $s_{2}$, R1 does not know the keys $s_{t}^{*}$ of the noisy tags and, therefore, cannot filter out $S_{t}^{*}$ to get *S* and compute ${\bar{s}}_{2}$. In particular, R1 knows $C_{eav}\cdot n/C_{s}=\left(1-C_{s}\right)\cdot n/C_{s}$ bits of *S*, but the remaining *n* bits remain unknown. Thus, once the KUP is completed, R1 has no control over the tag T and cannot trace it.

## 6. Conclusions

Cryptographic protection is usually handled at the application layer and cannot exploit signal features at the physical layer, which restricts its scope. We have shown in this paper that backward privacy of an OTP can be guaranteed with the use of channels with positive secrecy capacity. The implementation of such channels with noisy tags has been analyzed and the value $n_{T}=3$, for which the capacity of the eavesdropper’s channel is only $C_{eav}=0.22$ bits, provides a good compromise between performances and the ease of implementation. We also defined a communication model for RFID ownership transfer that captures spatiotemporal requirements. Protocols defined in this model can be applied to a wider range of practical scenarios. Finally, we have presented the first example of a symmetric-key OTP that does not require a TTP or ISE and formally proved that it is correct and secure in this model.

## Figures and Tables

**Figure 6:**
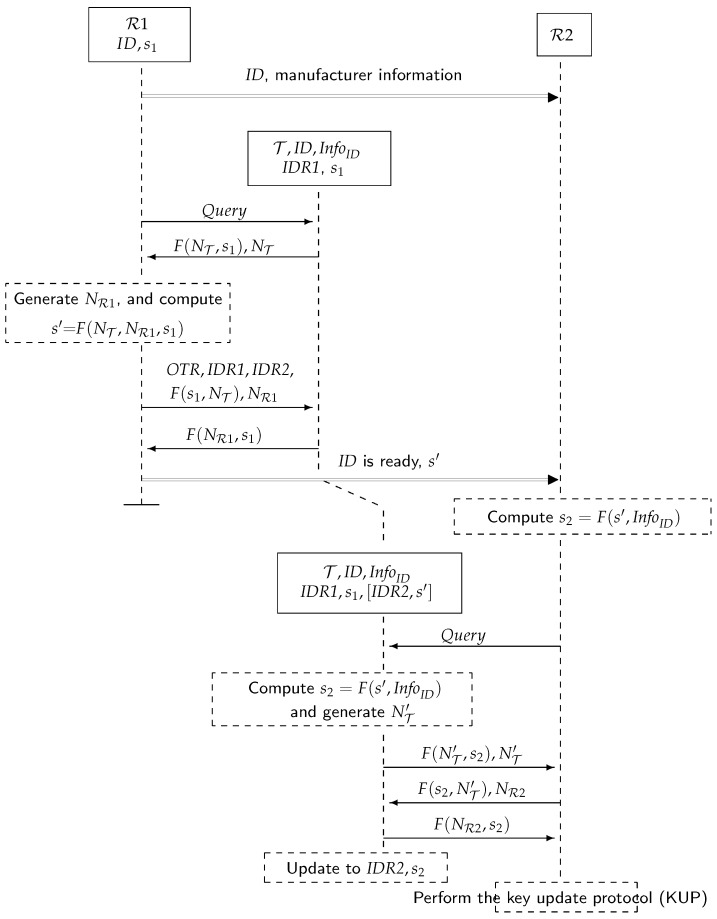
The ownership transfer protocol.

**Figure 7:**
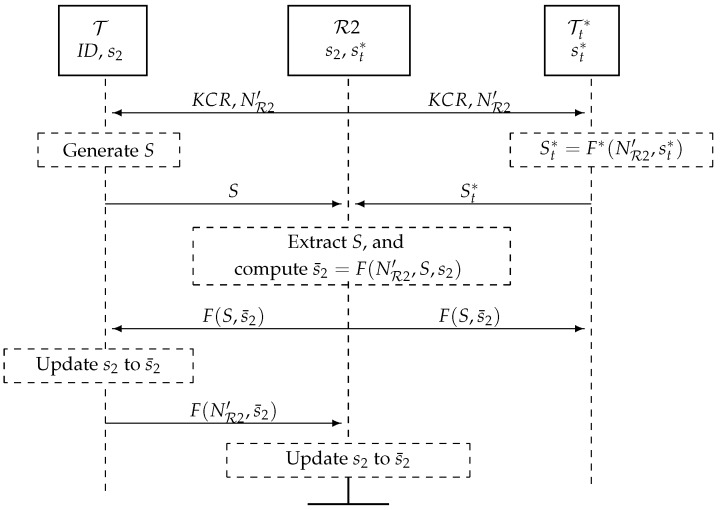
Key update protocol (KUP) with noisy tags $T_{t}^{*}$, $1\leq t\leq n_{T}$.

**Figure A1:**
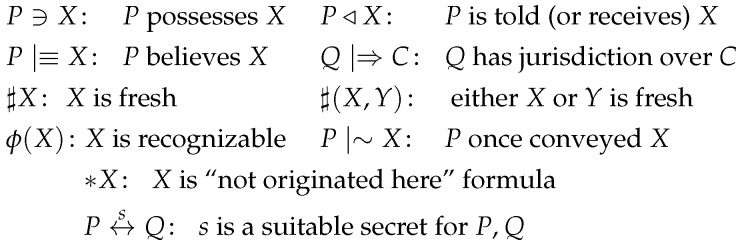
GNY reasoning notation.

**Figure A2:**
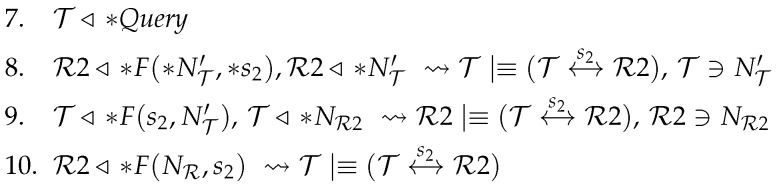
The parsed ownership transfer (OT) subprotocol.

**Figure A3:**
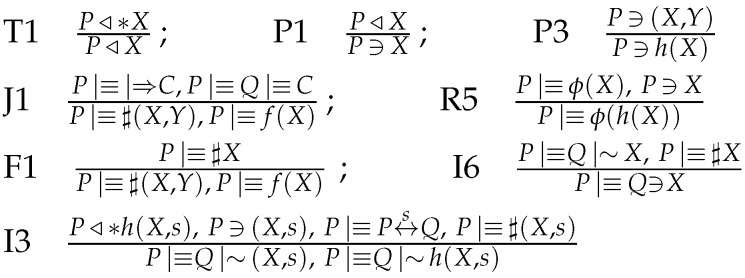
GNY logic postulates.

**Figure A4:**
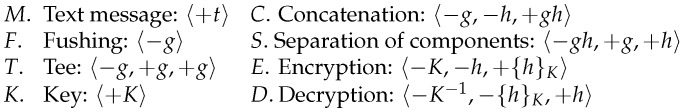
Atomic penetrator traces.

## Appendix A. Protocol Analysis

Because of space limitations, we only show here the consistency and correctness of the ownership transfer (OT) subprotocol in Section 5.1 (Flow 7–Flow 10). The analysis for the first part is similar.

### Appendix A.1. GNY Logic

In Figure A1, we present the notation we shall use: P,Q,… are protocol parties (principals); X,Y,… are formulae; and *s* is a key. The conjunction (X,Y) is also a formula.

Initial assumptions: At the beginning of each run of the OT subprotocol, we assume that parties T and R2: (i) believe (trust) each other: T∣≡R2∣≡T; (ii) believe that the secret $s_{2}$ to be shared between them is suitable: $T|\;\equiv (T\overset{s_{2}}{↔}R2)$ and $R2|\;\equiv (T\overset{s_{2}}{↔}R2)$; and (iii) believe in the jurisdiction of R1 over the secret $s_{2}$: $T|\;\equiv R1|\;\Rightarrow \left(T\overset{s_{2}}{↔}R2\right)\;\mathrm{and}\;R2|\;\equiv R1|\;\Rightarrow \left(T\overset{s_{2}}{↔}R2\right).$ In addition, each party possesses the secret key $s_{2}$ and a fresh nonce: $T∋s_{2},\;T∋N_{T}^{\prime },\;T|\;\equiv ♯N_{T}^{\prime },\;R2∋s_{2}$ and R2∋NR2, $R2|\;\equiv ♯N_{R2}\;.$ Finally, T believes that $\left(s_{2},N_{T}^{\prime }\right)$ is recognizable, and R2 believes that $\left(N_{T}^{\prime },s_{2}\right)$ and $\left(N_{R2},s_{2}\right)$ are recognizable: $T|\;\equiv \phi \left(s_{2},N_{T}^{\prime }\right),\;R2|\;\equiv \phi \left(N_{T}^{\prime },s_{2}\right)\;and\;R2|\;\equiv \phi \left(N_{R2},s_{2}\right).$

The goal of the OT subprotocol is for R2 and T to exchange the key $s_{2}$. The GNY logic parses the description of protocols for formal reasoning. A formalized description of the OT subprotocol is presented in Figure A2.

In this, Flows 8, 9 and 10 include message extensions (⋯⇝X) that are assumed assumptions. To prove consistency, we must show that on completion of the subprotocol, the following formulae can be deduced: $T∋s_{2}$, $T|\;\equiv (T\overset{s_{2}}{↔}R2)$, $T|\;\equiv R2∋s_{2}$, $R2∋s_{2}$, $R2|\;\equiv (T\overset{s_{2}}{↔}R2)$, $R2|\;\equiv T∋s_{2}$.

Four of these are initial assumptions. Therefore, we only need to show the formulae:

(A1) $$T\;|\;\equiv \;R2\;∋\;s_{2}\;,\;\;\mathrm{and}$$

(A2) $$R2\;|\;\equiv \;T\;∋\;s_{2}.$$

For this purpose, we use the deduction rules of GNY logic. A deduction rule consists of a set of premises $P_{1},\ldots ,P_{n}$ and a conclusion *C*, written: $\frac{P_{1},\;\ldots \;,P_{n}}{C}$. In Figure A3, we list the rules that we shall use to deduce formulae: f(X) and h(X) are computationally feasible functions of *X*, with h(X) a one-way function.

To show that Formulas (A1) and (A2) can be deduced from protocol assumptions and transmitted messages, we analyze below the parsed OT subprotocol in Figure A2.

7. No belief or possession can be derived from this message.
8. Apply the being-told rule T1 and the possession rule P1 to $R2\;◃\;*N_{T}^{\prime }$ to get $R2∋N_{T}^{\prime }$. Apply the recognizability rule R5 to the initial assumptions $R2|\;\equiv \phi (N_{T}^{\prime },s_{2})$ to get that R2 recognizes T. No postulate enables us to further derive new beliefs or possessions from this message. In particular, we cannot derive the freshness of the message.
9. Apply rules T1 and P1 to $T◃*N_{R2}$ to get $T∋N_{R2}$. Apply the freshness rule F1 to the initial assumptions $T|\;\equiv ♯N_{T}^{\prime }$, $\phi (s_{2},N_{T}^{\prime })$ to get $T|\;\equiv ♯(s_{2},N_{T}^{\prime })$. Apply the interpretation rule I3 to: the previous result, $T◃*\;F(s_{2},N_{T}^{\prime })$ and the initial assumptions $T∋(s_{2},N_{T}^{\prime })$ and $T|\;\equiv (T\overset{s_{2}}{↔}R2)$, to get $T|\;\equiv R2|\;∼s_{2}$. Now, apply rule I6 to get Formula (A1): $T|\;\equiv R2∋s_{2}$.
10. Apply the freshness rule F1 to the initial assumptions $R2|\;\equiv ♯N_{R2}^{\prime }$, $\phi (N_{R2}^{\prime },s_{2})$ to get $R2|\;\equiv ♯(N_{R2}^{\prime },s_{2})$. Apply rule I3 to: the previous result, $R2◃*\;F(N_{R2}^{\prime },s_{2})$ and the initial assumptions $R2∋(N_{R},s_{2})$ and $R2|\;\equiv (T\overset{s_{2}}{↔}R2)$, to get $R2|\;\equiv T|\;∼s_{2}$. Now, apply rule I6 to get Formula (A2): $R2|\;\equiv T∋s_{2}$.

It follows that the OT subprotocol is consistent. In particular,

(a) Possession consistency: transmitted messages only include formulae that the sender possesses;
(b) Belief consistency: message extensions include only beliefs held by the sender at the time he/she sends the message.

Strand spaces: We next show the correctness of the OT subprotocol using strand spaces [12,15]. To simplify the analysis, we remove Flow 7, which does not provide any cryptographic information.

A strand space Σ is a collection of strands and a graph generated by a causality relation. A strand *s* is a sequence of events that represent either a protocol execution by a legitimate party (principal) or a sequence of actions by a penetrator. We refer to the messages that can be exchanged between the principals as terms of the strand. In a protocol, principals can either send or receive terms, and this is represented with a positive or a negative sign, respectively. We write a⊏b if *a* is a subterm of *b*. The trace tr(s) of a strand is the sequence of its signed terms. A node of Σ is a pair n=〈s,i〉, with s∈Σ, 1≤i≤length(tr(s)). The set of nodes is denoted by N. We say that node n=〈s,i〉 belongs to strand *s*. term(n) is the *i*-th signed term $\mathrm{tr}{\left(s\right)}_{i}$ of *s*.

We write $n_{1}≺n_{2}$ to indicate that $n_{1}$ precedes $n_{2}$ in a strand (not necessarily immediately). An unsigned term *t* occurs in *n* iff t⊏term(n); *n* is an entry point for a set of terms I⊂T iff (if and only if) term (n)=+t for some t∈I, and whenever $n^{\prime }≺n$, then term $(n^{\prime })\notin I$. An unsigned term *t* originates on *n* iff *n* is an entry point for $I=\{t^{\prime }:t⊏t^{\prime }\}$. *t* is uniquely originating iff *t* originates at a unique n∈N. A bundle is a portion of a strand space that consists of strands of a protocol session that are hooked together, where one strand sends a message and the other receives the same message. For a protocol to be correct, each such bundle must contain one strand for each one of the legitimate principals participating in a session, with all parties agreeing on nonces and session keys. The penetrator (adversary) has a set of keys $K_{P}$ (shared with accomplices or “lost”) and a set of penetrator traces P that model her/his capabilities. Penetration traces typically require hooking several atomic traces. In Figure A4, we list the atomic penetrator traces we shall consider [12]. A protocol attack is captured by combining penetrator traces with protocol strands.

**Definition** **A1.** (Σ,P) *is an infiltrated strand space if* Σ *is a strand space and* P⊂Σ *is such that tr(p) is a penetrator trace for all* p∈P

**Definition** **A2.** *An infiltrated strand space* (Σ,P) *is an OTP space if* Σ *has three kinds of strands:*

Step 1. *Penetrator strands* s∈P
Step 2. *Initiator strands* $s\in Init[T,R2,N_{T}^{\prime },N_{R2}^{\prime }]$ *defined by:*
  $$\langle +\left(F\left(N_{T}^{\prime },s_{2}\right),N_{T}^{\prime }\right),-\left(F\left(s_{2},N_{T}^{\prime }\right),N_{R2}^{\prime }\right),+F\left(N_{R2}^{\prime },s_{2}\right)\rangle ,$$
  *with* $s_{2}\in K$, $N_{T}^{\prime },N_{R2}^{\prime }\notin K$. T *is the principal associated with this strand*.
Step 3. *Responder strands* $s\in Resp[T,R2,N_{T}^{\prime },N_{R2}^{\prime }]$, *defined by*:
  $$\langle -\left(F\left(N_{T}^{\prime },s_{2}\right),N_{T}^{\prime }\right),+\left(F\left(s_{2},N_{T}^{\prime }\right),N_{R2}^{\prime }\right),-F\left(N_{R2}^{\prime },s_{2}\right)\rangle ,$$
  *with* $s_{2}\in K$, $N_{T}^{\prime },N_{R2}^{\prime }\notin K$. R2 *is the principal associated with this strand*.

(A) Agreement: the responder’s guarantee:
**Proposition** **A1.** *Suppose that:* (Σ,P) *is an OTP space*, C *a bundle of* Σ, $s\in Resp[T,R2,N_{T}^{\prime },N_{R2}^{\prime }]$, $s_{2}\notin K_{P}$ *and* $N_{T}^{\prime }\neq N_{R2}$ *with* $N_{R2}$ *uniquely originating in* Σ. *Then*, C *contains an initiator strand* $t\in Init[T,R2,N_{T}^{\prime },N_{R2}]$.
**Proof.** We prove this using four lemmas. Let $n_{0}$ be the node 〈s,2〉 (the second node of the reader) that outputs the term $v_{0}=\left(F\left(s_{2},N_{T}^{\prime }\right),\;N_{R2}\right)$ and $n_{3}$ the node 〈s,3〉 that receives the term $v_{3}=F\left(N_{R2},s_{2}\right)$. Two additional nodes $n_{1},n_{2}$ such that $n_{0}≺n_{1}≺n_{2}≺n_{3}$ will be identified.
**Lemma** **A1.** $N_{R2}$ *originates at node* $n_{0}$.
**Proof.** We know that $N_{R2}⊏v_{0}$, and the sign of $n_{0}$ is positive. We just need to show that $N_{R2}\;⊏\;/\;\langle s,1\rangle$. Since term ($\langle s,1\rangle )=(F\left(N_{T}^{\prime },s_{2}\right),N_{T}^{\prime }$), we only need to check that $N_{T}^{\prime }\neq N_{R2}$, which is a hypothesis, and that $s_{2}\neq N_{R2}$, which follows from the stipulation $N_{R2}\notin K$. ☐

The next lemma establishes that the crucial step is taken by a regular strand and not a penetrator strand.
**Lemma** **A2.** *The set* $S=\{n\in C\;:v_{3}\;⊏\;term\left(n\right)\;\;\wedge \;\;\;v_{0}\;⊏\;/\;term\left(n\right)\}$ *has a* ⪯-minimal *node* $n_{2}$, *which is regular and has a positive sign*.
**Proof.** *S* is non-empty because $n_{3}\in C$; and $n_{3}$ contains $v_{3}$, but not $v_{0}$. Since *S* is a partially-ordered set (because C is), it has at least one ⪯-minimal node $n_{2}$, and its sign must be positive. Therefore, we just need to check that $n_{2}$ does not lie on a penetrator strand *p*. For this purpose, we shall examine all of the atomic penetrator traces tr(p) listed in Figure A4.

M. tr(p)=〈+t〉: Then, $N_{R2}⊏t$ and $N_{R2}$ originates on *t*, which is not possible because $N_{R2}$ originates on the regular node $n_{0}$ (Lemma A1).
F. tr(p)=〈-g〉: This has no positive nodes.
T,C tr(p)=〈-g,+g,+g〉 or 〈-g,-h,+gh〉: then, the positive nodes are not minimal occurrences.
K. tr$\left(p\right)=\langle +K_{0}\rangle$ with $K_{0}\in K_{P}$: Since $v_{3}⊏\;/\;K_{0}$, this case does not apply.
E. tr$\left(p\right)=\langle -K_{0},-h,+{\left\{h\right\}}_{K_{0}}\rangle$: Suppose $v_{3}⊏{\left\{h\right\}}_{K_{0}}$. Then, $h=N_{R2}$, $K_{0}=s_{2}$. Thus, there is a node *m* (the first of this strand) with term ($m)=s_{2}$. However, $s_{2}\notin K_{P}$, so that this node is regular, but no regular node originates $s_{2}$. This contradicts the initial assumption.
D. tr$\left(p\right)=\langle -K_{0}^{-1},-{\left\{h\right\}}_{K_{0}},+h\rangle$: If the positive node is minimal in *S*, then $v_{0}⊏\;/\;h$ and $v_{0}⊏{\left\{h\right\}}_{K_{0}}$. However, because $v_{0}\neq {\left\{h\right\}}_{K_{0}}$, if $v_{0}⊏{\left\{h\right\}}_{K_{0}}$, then $v_{0}⊏h$, which is a contradiction.
S. tr(p)=〈-gh,+g,+h〉: Assume term $(n_{2})=h$ (there is a symmetric case with term $(n_{2})=g$). By the minimality of $n_{2}$, $v_{0}⊏gh$. Hence, $g=F(N_{T}^{\prime },s_{2})$ and $h=N_{R2}$. However, then $v_{3}⊏\;/\;h$ and $n_{2}\notin S$, contradicting the initial assumption.

Therefore, $n_{2}$ does not lie on a penetrator strand. ☐
**Lemma** **A3.** *Node* $n_{2}$ *follows* $n_{1}$ *on the same regular strand t, and term* $\left(n_{1}\right)=\left(F\left(s_{2},N_{T}^{\prime }\right),N_{R2}\right)$.
**Proof.** From Lemma A1, we know that $N_{R2}$ originates at $n_{0}$, and by assumption, it is unique in Σ. Furthermore, $n_{2}\neq n_{0}$ since $v_{0}⊏$ term $\left(n_{0}\right)$ and $v_{0}⊏/$−1.5 mm term $\left(n_{2}\right)$. Therefore, $N_{R2}$ does not originate at $n_{2}$, and there is a node $n_{1}$ preceding $n_{2}$ on the same strand, such that $N_{R2}⊏$ term $\left(n_{1}\right)$. By the minimal property of $n_{2}$, $v_{0}⊏$ term $\left(n_{1}\right)$. However, as no regular node contains a combination as a proper subterm, $\mathrm{term}\left(n_{1}\right)=\left(F\left(s_{2},N_{T}^{\prime }\right),N_{R2}\right)$. ☐
**Lemma** **A4.** The regular strand t containing $n_{1}$ *and* $n_{2}$ *is an initiator strand contained in* C.
**Proof.** $n_{1}$ precedes $n_{2}$ in the same strand. Node $n_{2}$ is a positive regular node and comes after a node with the form $\left(F\left(s_{2},N_{T}^{\prime }\right),N_{R2}\right)$. Hence, *t* is an initiator strand, since a responder strand would only contain a negative node after one of that form. Thus, $n_{1}$ and $n_{2}$ are the second and the third nodes of *t*, respectively. ☐ Lemmas A3 and A4 complete the proof of Proposition A1. ☐

**Proposition** **A2.** *If* (Σ,P) *is an OTP space and* $N_{T}^{\prime }$ *is uniquely originating in* Σ, *then there is at most one strand* $t\in Init[T,R2,N_{T}^{\prime },N_{R2}^{\prime }]$ *for any* T, R2 *and* $N_{R2}$.

**Proof.** Let $t\in \mathrm{Init}[T,R2,N_{T}^{\prime },N_{R2}]$ for T, R2 and $N_{R2}$. Then, 〈t,1〉 is positive, $N_{T}^{\prime }⊏\mathrm{term}\langle t,1\rangle$, and $N_{T}^{\prime }$ cannot possibly occur earlier on *t*. Therefore, $N_{T}^{\prime }$ originates at node 〈t,1〉. Since $N_{T}^{\prime }$ originates uniquely in Σ, there can be at most one such *t*. ☐

(B) Agreement: the initiator’s guarantee:
**Proposition** **A3.** *Suppose that*: (Σ,P) *is an OTP space*, C *is a bundle of* Σ, $s\in Init[T,R2,N_{T}^{\prime },N_{R2}]$, $s_{2}\notin K_{P}$ *and* $N_{T}^{\prime }$ *is uniquely originating in* Σ. *Then, there exists a responder strand* $t\in Resp[T,R2,N_{T}^{\prime },N_{R2}]$.
**Proof.** Consider the set $\left\{m\in C\;:F\left(s_{2},N_{T}^{\prime }\right)\;⊏\;\mathrm{term}\left(m\right)\right\}$. This is not empty, because it contains 〈s,2〉, and so, it contains a minimal node $m_{0}$. If $m_{0}$ lies on a regular strand *t*, then we can show that $t\in \mathrm{Resp}[T,R2,N_{T}^{\prime },N_{R2}]$. If instead, $m_{0}$ lies on a penetrator strand *p*, then *p* should be an E-strand with trace: $\langle -s_{2},-N_{T}^{\prime },+F\left(s_{2},N_{T}^{\prime }\right)\rangle$, but this contradicts the assumption $s_{2}\notin K_{P}$. ☐
